# Variation in Rubisco activase (*RCAβ*) gene promoters and expression in soybean [*Glycine max* (L.) Merr.]

**DOI:** 10.1093/jxb/ert346

**Published:** 2013-10-29

**Authors:** Maoni Chao, Zhitong Yin, Derong Hao, Jinyu Zhang, Haina Song, Ailing Ning, Xiaoming Xu, Deyue Yu

**Affiliations:** ^1^National Center for Soybean Improvement, National Key Laboratory of Crop Genetics and Germplasm Enhancement, Nanjing Agricultural University, Nanjing 210095, China; ^2^Jiangsu Provincial Key Laboratory of Crop Genetics and Physiology, Yangzhou University, Yangzhou 225009, China; ^3^Jiangsu Yanjiang Institute of Agricultural Sciences, Nantong 226541, China; ^4^Photosynthesis Research Laboratory, College of Life Sciences, Nanjing Agricultural University, Nanjing 210095, China

**Keywords:** Allele mining, eQTL, photosynthesis, promoter, Rubisco activase, seed yield, soybean [*Glycine max* (L.) Merr.]

## Abstract

Understanding the genetic basis of Rubisco activase (RCA) gene regulation and altering its expression levels to optimize Rubisco activation may provide an approach to enhance plant productivity. However, the genetic mechanisms and the effect of *RCA* expression on phenotype are still unknown in soybean. This work analysed the expression of RCA genes and demonstrated that two RCA isoforms presented different expression patterns. Compared with *GmRCAα*, *GmRCAβ* was expressed at higher mRNA and protein levels. In addition, *GmRCAα* and *GmRCAβ* were positively correlated with chlorophyll fluorescence parameters and seed yield, suggesting that changes in expression of *RCA* has a potential applicability in breeding for enhanced soybean productivity. To identify the genetic factors that cause expression level variation of *GmRCAβ*, expression quantitative trait loci (eQTL) mapping was combined with allele mining in a natural population including 219 landraces. The eQTL mapping showed that a combination of both *cis*- and *trans*-acting eQTLs might control *GmRCAβ* expression. As promoters can affect both *cis*- and *trans*-acting eQTLs by altering *cis*-acting regulatory elements or transcription factor binding sites, this work subsequently focused on the promoter region of *GmRCAβ*. Single-nucleotide polymorphisms in the *GmRCAβ* promoter were identified and shown to correlate with expression level diversity. These SNPs were classified into two groups, A and B. Further transient expression showed that GUS expression driven by the group A promoter was stronger than that by the group B promoter, suggesting that promoter sequence types could influence gene expression levels. These results would improve understanding how variation within promoters affects gene expression and, ultimately, phenotypic diversity in natural populations.

## Abstract (precis)

Variation in Rubisco activase gene promoters (*RCAβ*) could affect diversity of expression level, which provided a new approach for enhancing soybean productivity by altering the expression level of RCAβ

## Introduction

Soybean [*Glycine max* (L.) Merr.] is the world’s most widely grown legume, and it provides high levels of protein, oil, and other nutrients for humans. The future demand for soybean will increase not only as the world population increases but also as incomes improve and diets become more meat intensive (Ainsworth *et al.*, 2012). As crop productivity gains through traditional breeding begin to lag, novel strategies for improving crop yield potential have begun to examine supercharging photosynthesis to drive a new ‘green revolution’ (Raines, 2011).

Rubisco activase (RCA), which catalyses the activation of the key photosynthesis enzyme Rubisco (Portis, 2003), has been identified as one of several new potential targets for improving plant productivity and seed yield (Yin *et al.*, 2010b). In several plant species, there are two closely related forms of RCA proteins, with molecular masses of 41kDa (β-isoform) and 47kDa (α-isoform), although tobacco only has the β-isoform (Salvucci *et al.*, 1987). These two isoforms are capable of activating Rubisco (Salvucci *et al.*, 2003), but they also have physiological significance in thermal sensitivity under heat stress conditions (Sanchez de Jimenez *et al.*, 1995; Crafts-Brandner and Salvucci, 1997, 2004; Law and Crafts-Brandner, 2001; Portis, 2003). Studies on rice (Wang *et al.*, 2010) and spinach (Crafts-Brandner *et al.*, 1997) demonstrated that the α-isoform was more thermostable than the β-isoform, suggesting that the α-RCA isoform may play an important role in photosynthetic acclimation to moderate heat stress *in vivo*, whereas the β-RCA isoform was shown to play a major role in maintaining Rubisco’s initial activity under normal conditions (Wang *et al.*, 2010). Understanding the genetic basis of RCA gene regulation and the differential expression of the two isoforms may provide a mechanism for optimizing Rubisco activation under prevailing environmental conditions (Crafts-Brandner *et al.*, 1997).

In contrast to the RCA gene and its encoded protein, which have been well studied in a number of species, the molecular mechanism of transcriptional regulation about RCA gene has thus far been only studied in spinach (Orozco and Ogren, 1993), *Arabidopsis* (Liu *et al.*, 1996), potato (Qu *et al.*, 2011), and rice (Yang *et al.*, 2012). Gene transcription is a primary intermediate between the information encoded in the genome and the final phenotype. In this context, variation in expression level may be an important cause of phenotypic variation (Alonso-Blanco *et al.*, 2009; Bullard *et al.*, 2010). A previous study demonstrated that many phenotypic differences between species may be due to alteration in gene expression regulation rather than protein structure (King and Wilson, 1975). Recent studies on soybean RCA genes have shown that the expression of the two isoforms was positively correlated with Rubisco activity, photosynthetic rate, and seed yield (Yin *et al.*, 2010b). Thus, an analysis of the determinants of *RCA* expression not only helps in understanding the genetic basis of *RCA* regulation but also provides an approach to improve soybean photosynthesis and productivity by altering *RCA* expression levels to optimize Rubisco activation under the prevailing environmental conditions.

Expression quantitative trait locus (eQTL) mapping has been used to identify loci that might affect gene expression in a *cis*- or *trans*-acting manner (Delker and Quint, 2011). However, the nature of the sequence variation in the eQTLs that cause expression changes associated with phenotypic variation is a key and usually unknown feature for researchers and breeders (Vuylsteke and van Eeuwijk, 2008). Currently, mining for sequence variation is gaining more importance in the context of gene expression (Kumar *et al.*, 2010). When a eQTL is *cis*-acting, *cis*-regulatory polymorphisms may affect the gene expression level in an allele-specific manner by altering *cis*-acting regulatory elements in the promoter or by modifying target sites for mRNA processing and stability in the 3′-untranscribed regions (3′-UTR), whereas *trans*-acting polymorphisms modify the sequence polymorphisms of the transcription factor or transcription factor-binding sites at *cis*-regulatory sequences in an allele-specific manner for both alleles, causing expression changes in *trans*-acting factors (Vuylsteke and van Eeuwijk, 2008). Interestingly, both types of polymorphisms are associated with regulatory sequence variations of the promoter, which affect *cis*-regulatory polymorphisms and *trans*-acting polymorphisms by altering the 5′-upstream *cis*-acting regulatory elements or transcription factor-binding sites. This work attempted to use a new strategy that combines eQTL mapping and allele mining in a natural population to understand the genetic basis of RCA gene regulation in soybean.

Both of the genes encoding the larger isoform (*GmRCAα*) and smaller isoform (*GmRCAβ*) in soybean have been cloned and characterized (Yin *et al.*, 2010b). However, the expression pattern, genetic basis of the genes’ regulation, and the correlation between *RCA* expression and phenotype under natural conditions remained unknown. In this study, further analysis found that different patterns of RCA isoform expression were present in soybean, which may be necessary to control enzyme activity and increase overall soybean photosynthesis and productivity. In addition, both genes had a positive correlation with soybean photosynthesis and productivity under natural conditions. To understand the genetic basis of *GmRCAβ* gene regulation, a new strategy was used to identify the single-nucleotide polymorphisms (SNPs) that determine the expression level by combining eQTL mapping and allele mining in a natural population. All SNPs associated with *GmRCAβ* gene expression existed in a linked manner with the same *P*-value, indicating that these SNPs might affect gene expression together, and any of the SNPs might be used as an allele-specific molecular marker (Kumar *et al.*, 2010) to optimize gene expression. These allele-specific molecular markers would contribute to understanding the molecular basis of the genetic regulation of gene expression and facilitate the introgression of the novel alleles through marker-assisted breeding.

## Materials and methods

### Plant materials and plant growth conditions

Soybean Kefeng No. 1 was grown under natural conditions in the field at Nanjing Agricultural University. Extracts prepared from the plants were used for immunoblot and expression pattern analysis of the RCA gene. Sowing was done on 17 June 2011. Once the upper-third leaves had fully expanded, they were collected and frozen immediately in liquid nitrogen for Western blot analysis. Leaves, stems, roots, and flowers collected at the flowering stage (R2 stage) and seeds harvested at the full seed stage (R6 stage) were used for the gene expression analysis. At the R2 stage, the mature upper-third leaves were collected individually from three plants at different time points during a 48-h cycle for analysis of the diurnal pattern of *RCA* expression.

A panel of 219 soybean landraces collected at 53–24 ° N 134–97 ° E in China was used to evaluate RCA gene expression, empirical chlorophyll transient parameters, seed weight, and seed yield. Of the 219 landraces, 191 landraces that had been genotyped for 1142 SNPs with minor allele frequencies of higher than 10% were used for the association analysis. These landraces originated from 24 provinces in China and covered four ecological regions: northeast region, north region, Huanghuai region, and south region (Supplementary Fig. S1, available at *JXB* online). These four regions represented the four major planting areas of soybean in China (Li *et al.*, 2008). Seeds of all landraces were obtained from germplasm storage in the National Center for Soybean Improvement (Nanjing, China). The trials were performed under natural conditions at Jiangpu Experimental Station, Nanjing Agricultural University. All landraces were grown using previously described procedures (Yin *et al.*, 2010b).

To control the environmental effects on phenotypic evaluation, the natural population was divided into four groups according to their time to maturity as observed in previous years (data not shown). Each group was sown at different times so that when trait data were collected, all landraces were at a similar growth stage. Sowing was done on 7, 17, and 27 June and 7 July 2011. Nutrition and water were supplied sufficiently throughout the experiment to avoid potential nutrient and drought stresses. At the R6 stage of development, the mature upper-third leaves were collected individually from three plants of each landrace in the morning (09:00–11:30) on a sunny day, frozen immediately in liquid nitrogen, and stored at –80 °C. In order to reduce systematic errors caused by the time of sampling, time segment was taken as the block, which represented one repetition of each landrace, and sampling of each block by three groups of people was accomplished in a short period of time (less than 50 minutes) to reduce the effect of time of sampling on each material within block. The variance analysis of *GmRCAα* and *GmRCAβ* expression is shown in Supplementary Table S1.

### RNA isolation and synthesis of cDNA

Total RNA was isolated from leaves using a RNA Plant Extraction Kit (Tiangen, China) and treated with RNase-free DNase I (Takara, Japan) to remove contamination by genomic DNA. Approximately 2 µg of purified total RNA was reverse transcribed using AMV reverse transcriptase (Takara) with oligo-dT-18 as the primer (Takara), according to the manufacturer’s instructions.

### DNA sequencing and genotyping

Genomic DNA was extracted from the young leaves of soybean plants using a DNA plant extraction kit (Tiangen). The *GmRCAβ* promoter was amplified from genomic DNA with the following primers: sense 5′-TGGCAGTAGCTGTTTCTAGTGATGG-3′ and anti-sense 5′-GCCTACTCGTTTTTACATCCCCTTA-3′. PCR was conducted in a 50-µl reaction volume using KOD polymerase (Toyobo, Japan) following the manufacturer’s recommendations, using a PTC-225 thermal cycler (MJ Research, Watertown, MA, USA). The cycling program consisted of one cycle at 94 °C for 2min, 35 cycles at 94 °C for 15 s and 68 °C for 3min, and one cycle at 4 °C for 10min. Amplification products were separated by electrophoresis on 1% agarose, and the band of expected size was excised and purified with a PCR gel purification kit AP-GX-4 (Axygen, China). Three sample PCR products were purified from soybean landraces and sequenced at Invitrogen (Shanghai, China). All sequences were checked manually and all singletons observed were verified by sequencing newly amplified fragments. Then the sequence was aligned using CLUSTALX version 1.83 (Thompson *et al.*, 1997). A manual check was performed in every case to ensure sequencing and alignment quality. Polymorphism data was analysed using DnaSP version 4.10 (Rozas *et al.*, 2003) to identify sequence variation. Identified SNPs with a minor allele frequency at least 10% were genotyped in the natural population used for the candidate-gene association analysis.

### Bioinformatics analysis

Multiple sequence alignment was performed using CLUSTALX version 1.83. Phylogenetic trees were constructed using both neighbour-joining and Bayesian approaches. In the neighbour-joining method, the phylogenetic analysis was conducted using MEGA version 4.1(Kumar *et al.*, 2008). The parameter setups were as follows: model = p-distance; bootstrap = 1000 replicates; and gap/missing data = pairwise deletion. In the Bayesian method, the analyses were conducted using MrBayes version 3.1 (Ronquist and Huelsenbeck, 2003) with the Jones, Taylor, Thornton substitution model (Jones *et al.*, 1992), four chains, 1 million generations, and two runs. Trees were sampled every 100 generations, discarding a burn-in of 250 000 generations. The putative *cis*-acting regulatory elements were identified through search against both the PlantCARE and PLACE databases (Prestridge, 1991; Higo *et al.*, 1999; Lescot *et al.*, 2002).

### Vector construction and *Agrobacterium*-mediated transformation

Two types of promoter sequences of *GmRCAβ* (type 2 and type 4) which belonged to promotor group A and group B, respectively (see Results) were cloned in soybean Suxie No. 1 and Kefeng No. 1, respectively. These 2300-bp fragments were fused with the β-glucuronidase (GUS) gene as a reporter in the binary vector pCAMBIA1381Z (Cambia, Australia) digested with corresponding restriction enzymes. The binary vector constructs, promoterless control (pCAMBIA1381Z), and CaMV 35S promoter control (pCAMBIA1301) were transformed into *Agrobacterium tumefaciens* EHA105 by the freeze–thaw method. The constructs were introduced into the soybean cotyledonary node via *Agrobacterium*-mediated transformation following Zhang *et al.* (1999).

### Histochemical staining

GUS activity was determined in soybean cotyledonary nodes based on a previously described method (Jefferson *et al.*, 1987) with some modifications. Plant materials were immersed in 50mM sodium phosphate buffer (pH 7.0) containing 0.1% Triton X-100, 10mM EDTA, 0.5mg/ml 5-bromo-4-chloro-3-indolyl glucuronide, 1mM K_3_[Fe(CN)_6_], and 1mM K_4_[Fe(CN)_6_] for 24h at 37 °C in darkness. The staining solution was then removed and the samples were dehydrated using an ethanol series of 70, 80, 90, and 100%, with exposure to each for a minimum of 60min. GUS staining was observed under an Olympus SZX12 stereomicroscope and photographed with a digital camera (CoolSNAP, RS photometrics).

### Leaf protein extraction and Western blot analysis

For immunoblot analysis, the proteins were extracted using previously described procedures (Yin *et al.*, 2010b). Protein extracts were separated by 12% SDS-PAGE and then transferred onto a nitrocellulose membrane (Bio-Rad) as described by Mitsuhashi and Feller (1992) using a Trans Blot system (Bio-Rad). Then, the nonspecific binding of antibodies was blocked with 5% nonfat dried milk in phosphate-buffered saline (pH 7.4) for 2h at room temperature. The membranes were then incubated overnight at 4 °C with polyclonal anti-cotton RCA antibodies (AS10700, Agrisera) diluted to 1:10 000 in phosphate-buffered saline plus 1% nonfat milk. The immune complexes were detected using goat anti-rabbit IgG-horseradish peroxidase (sc-2004; Santa Cruz Biotechnology) and developed with a solution containing 3,3′-diaminobenzidine tetrahydrochloride as the peroxidase substrate and then the membranes were scanned.

### Trait measurement and phenotypic data collection

The data on seed weight and seed yield were collected at maturity. Seeds of six plants per genotype were hand harvested and dried to constant weight (Yin *et al.*, 2010b).

Empirical chlorophyll transient parameters, such as F_v_/F_m_, Φ_PSII_, qP, and NPQ (see Table 2 for definitions), were measured using the upper-third leaf of the soybean plants at the R2 development stage. All measurements were performed at 25 °C using a PAM fluorometer (PAM2100, Heinz Walz, Effeltrich, Germany) using a previously described procedure (Yin *et al.*, 2010a).

**Table 2.** Correlation coefficients among *GmRCAα* and *GmRCAβ* expression and yield components in a natural soybean population

Trait	F_v_/F_m_	Φ_PSII_	qP	NPQ	Seed weight	Seed yield
*GmRCAβ* expression	0.107	0.219*	0.319**	–0.042	0.180*	0.303**
*GmRCAα* expression	0.087	0.198*	0.304**	–0.031	0.138	0.251**

F_v_/F_m_, maximum quantum yield of PSII primary photochemistry in the dark-adapted state; Φ_PSII_, actual quantum yield in the light-adapted state; qP, photochemical quenching coefficient; NPQ, non-photochemical quenching parameter describing the regulated dissipation of excess energy. *, *P*<0.05; **, *P*<0.01.

Expression levels were determined by quantitative real-time PCR (qRT-PCR). Because of the high homology between *GmRCAα* and *GmRCAβ* (Yin *et al.*, 2010b), specific primers and Taq Man-MGB probe (ABI, America) were applied to detect the specific expression levels. Expression of the soybean endogenous reference gene *Tubulin* (GenBank accession number AY907703.1) was used to normalize the transcript level in each sample. The primers and Taq Man-MGB probe used were as follows: Tub-F (5′ GGAGTTCACAGAGGCAGAG-3′) and Tub-R (5′-CACTTACGCATCACATAGCA-3′) for *Tubulin*; RCAa*-*F (5′-GATGGGCGTATGGAGAAGTTCT-3′), RCAα-R (5′-TGCGGAAAATTCCATTGCA-3′), and RCAα-MGB (5′-ACG ATCGTGTTGGCG-3′) for *GmRCAα; RCAβ*-F (5′-GGGACCAG CTTGAAGAA GGTTA-3′), *RCAβ*-R (5′-TGCTGGGTCTCTTCA ATCTCTTT-3′), and *RCAβ*-MGB (5′-CAGCAAGGTTTCCGG TG-3′) for *GmRCAβ.*

### Statistical analysis and association mapping

Statistical analyses were performed using SAS system 9.0 for Windows and Microsoft Excel 2003. Analysis of variance was performed using SAS PROC GLM. The mean values of *RCA* expression for each landrace were calculated using SAS PROC MEANS. The regressive coefficients among the traits were calculated using SAS PROC REG. The Pearson phenotypic correlations among the traits were calculated using SAS PROC CORR. The significant differences in the gene expression levels of two RCA genes were determined using the two-tailed Student’s t-test of Microsoft Excel 2003, and differences at *P* ≤ 0.01 were considered significant. The association of each SNP in the promoter region with expression levels was examined by the candidate-gene association analysis method (Harjes *et al.*, 2008; Rafalski, 2010). eQTLs for the expression of *GmRCAβ* were identified using genome-wide association analysis with the same association mapping panel and the same method used in a previous study (Hao *et al.*, 2012).

## Results

### Expression and protein levels

**Fig. 1.**
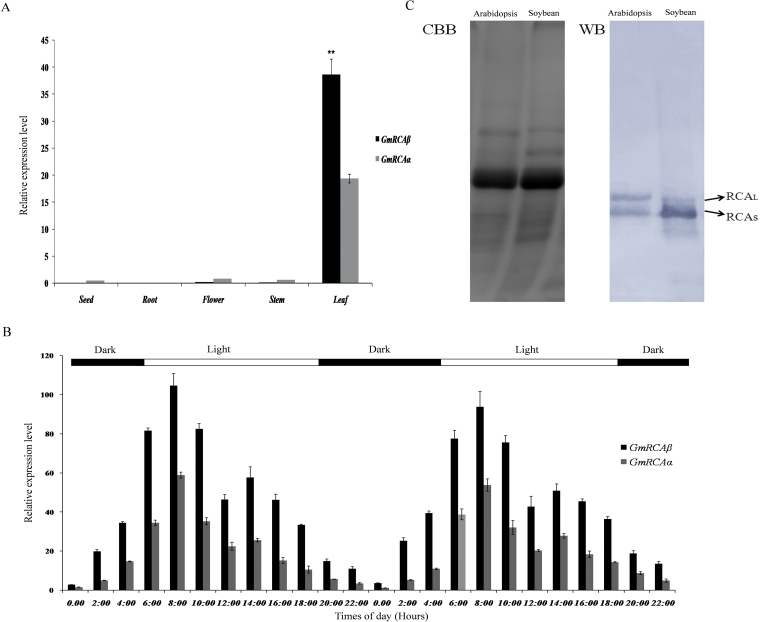
Expression pattern and Western blot analysis of *RCA* genes in soybean. (A) Quantitative real-time PCR analysis of organ-specific *RCA* expression in soybean; total RNA was isolated from flowers, roots, stems, and leaves harvested at R2 stage (flowering) and from seeds harvested at R6 stage (full seed); error bars represent standard errors of three independent repetitions. (B) Diurnal pattern of *RCA* mRNA accumulation in soybean leaves; total RNA was extracted at different time points during a 48-h cycle from the upper-third leaves of soybean at R2 stage; error bars represent standard errors of three independent repetitions. (C) Western blot analysis of the two RCA isoforms from *Arabidopsis* and soybean; extracts of total soluble protein from *Arabidopsis* and fully expanded young soybean leaves (4.2 µg) were separated by 12% SDS-PAGE; protein bands were detected by Coomassie blue staining (CBB) or Western blot (WB) probed with antibodies to RCA and visualized using alkaline phosphatase conjugated to a secondary antibody.

Quantitative real-time PCR analysis was conducted to investigate the expression of RCA genes in soybean. RCA expression was high in leaves where photosynthesis takes place, but low in flowers, stems, seeds, and roots (Fig. 1A). To examine whether RCA gene expression in soybean followed a diurnal pattern in addition to being affected in a tissue-specific manner, qRT-PCR analysis was performed with total RNA extracted from soybean leaves harvested at different time points during a 48-h cycle. The maximal level of RCA gene expression was detected at 08:00, 2h after the beginning of the light period. Following this peak in expression, the levels were significantly decreased at noon and then slightly increased in the early afternoon, but the overall decreasing tendency remained (Fig. 1B). The minimum observed levels were detected at midnight. These results suggested that the expression of *RCA* followed a diurnal pattern and was highest in the morning.

Because the two RCA isoforms in soybean are derived from separate genes, and are not produced by alternative splicing of a single RCA gene (Yin *et al.*, 2010b), they might differ in expression and protein levels. Thus, the current work investigated the correlation between the two RCA isoforms at the mRNA and protein levels. As shown in Fig. 1A and B, *GmRCAβ* mRNA accumulated to a higher level than *GmRCAα* mRNA. Further analysis indicated that the expression levels of *GmRCAα* and *GmRCAβ* were significantly different (Fig. 1A). To test whether the relatively low expression of *GmRCAα* was in line with changes in protein levels, Western blot analysis was conducted. The results indicated that soybean plants contained two isoforms, with a higher concentration of the small isoform (Fig. 1C). In contrast, *Arabidopsis* contained two isoforms in almost equal amount, as reported by other study (Salvucci *et al.*, 1987).

### *RCA* promoter sequences

The promoter sequences of *GmRCAα* and *GmRCAβ* were analysed to determine whether the two different expression levels could arise from the regulatory elements and promoter sequence differences. The alignment of the 2300-bp promoter sequences of *GmRCAα* and *GmRCAβ* showed that two sequences matched poorly, with only 45.2% identity (Supplementary Fig. S2). Bioinformatics analysis showed that several important *cis*-acting elements, including the elements responsive to light, biotic stress and phytohormone and other basal element in the upstream regulatory region were similar for the two promoters, while the specific *cis*-elements and their copy numbers were different between *GmRCAα* and *GmRCAβ* promoter (Table 1). Moreover, heat stress-responsive elements were observed in the promoter region of *GmRCAα* but not in *GmRCAβ*, which was consistent with the previous study that the isoforms have physiological significance in thermal sensitivity under heat-stress conditions (Sanchez de Jimenez *et al.*, 1995; Crafts-Brandner and Salvucci, 1997, 2004; Law and Crafts-Brandner, 2001; Portis, 2003). These results indicated that expression and regulation of *GmRCAα* and *GmRCAβ* was a complex process and that the difference in numbers of specific *cis*-elements and some common *cis*-elements might be the reason for the different expression of the two isoforms.

**Table 1.** Comparison of *cis*-acting elements in the promoter regions of *GmRCAα* and *GmRCAβ*

Element type	Name	Copy number in promoter		Motif sequence (5′–3′)	Function
		*GmRCAα*	*GmRCAβ*		
Light	GA motif	1	0	AAAGATGA	Part of a light-responsive element
	I-box	1	0	CTCTTATGCT	Part of a light-responsive element
		1	0	TATTATCTAGA	
	ATCT motif	1	0	AATCTAATCT	Part of a conserved DNA module involved in light responsiveness
	Sp1	1	0	CC(G/A)CCC	Light-responsive element
	TCT motif	1	0	TCTTAC	Part of a Light-responsive element
	chs-CMA1a	1	0	TTACTTAA	Part of a light-responsive element
	GTGGC motif	1	0	CATCGTGTGGC	Part of a light-responsive element
	AT1 motif	0	1	AATTATTTTTTATT	Part of a light-responsive module
	GAG motif	0	1	AGAGATG	Part of a light-responsive element
	GT1 motif	0	1	GGTTAA	Light-responsive element
	G-box	0	3	CACGTT	*cis*-Acting regulatory element involved in light responsiveness
		1	0	CACGTG	
		1	0	TACGTG	
		1	2	CACGTG	
		0	1	CACGTA	
		0	1	ACACGTGT	
	Box 4	3	3	ATTAAT	Part of a conserved DNA module involved in light responsiveness
	Box II	1	0	CCACGTGGC	Part of a light-responsive element
		0	1	ACACGTAGA	
Circadian	Circadian	2	2	CAAAGATATC	*cis*-Acting regulatory element involved in circadian control
Phytohormone					
	AuxRR core	0	1	GGTCCAT	*cis*-Acting regulatory element involved in auxin responsiveness
	TGA element	0	2	AACGAC	Auxin-responsive element
	TCA element	0	1	GAGAAGAATA	*cis*-Acting element involved in salicylic acid responsiveness
	P-box	0	1	CCTTTTG	Gibberellin-responsive element
	GARE motif	0	1	TCTGTTG	Gibberellin-responsive element
		1	0	AAACAGA	
	ABRE	1	0	TACGTG	*cis*-Acting element involved in the abscisic acid responsiveness
		1	2	CACGTG	
		1	0	ACGTGGC	
Biotic stress	HRE	2	0	AAAAAATTTC	*cis*-Acting element involved in heat stress responsiveness
	Box-W1	1	0	TTGACC	Fungal elicitor-responsive element
	MBS	0	1	CGGTCA	MYB binding site
		0	2	TAACTG	MYB binding site involved in drought inducibility
	ARE	0	1	TGGTTT	*cis*-Acting regulatory element essential for anaerobic induction
Basal element	CAAT box	23	21	CAATT/CAAT/CCAAT	Common *cis*-acting element in promoter and enhancer regions
	TATA box	42	45	TATA/ATATAT/TTTTA	Core promoter element around –30bp of transcription start
	5′-UTR pyrimidine-rich stretch	1	2	TTTCTTCTCT	*cis*-Acting element conferring high transcription levels
Other	CAT box	1	1	GCCACT	*cis*-Acting regulatory element related to meristem expression
	GCN4 motif	0	4	CAAGCCA	*cis*-Regulatory element involved in endosperm expression
	HD zip	0	1	CAAT(A/T)ATTG	Element involved in differentiation of the palisade mesophyll cells
		0	1	CAAT(G/C)ATTG	Element involved in the control of leaf morphology development
	Skn-1 motif	2	2	GTCAT	*cis*-Acting regulatory element required for endosperm expression
	ATGCAAAT motif	1	0	ATACAAAT	*cis*-Acting regulatory element associated to the TGAGTCA motif
	O2 site	1	0	GATGACATGA	*cis*-Acting regulatory element involved in zein metabolism regulation

ABRE, abscisic acid-responsive element; ARE, anaerobic induction- responsive element; GARE, GA-responsive element; HD zip, homeodomain leucine zipper; HRE, heat stress-responsive element; MBS, MYB binding site; UTR, untranscribed region.

### Correlation between gene expression and chlorophyll ﬂuorescence and yield in a natural population

Empirical chlorophyll transient parameters, such as F_v_/F_m_, Φ_PSII_, qP, and NPQ, have been widely accepted as reflecting the structure and function of the photosynthetic apparatus (Papageorgiou, 2004). To further investigate the relationship between the effect of RCA gene expression on soybean photosynthesis and productivity, gene expression, chlorophyll ﬂuorescence parameters, and yield components was measured in a natural population. As shown in Supplementary Table S1, the expression levels of the two *RCA* genes displayed high diversity in soybean germplasm, ranging from 0.47 to 66.41 for *GmRCAα* expression and from 2.38 to 196.49 for *GmRCAβ* expression. Variation analysis indicated that these wide ranges might mainly be caused by genotypic differences. Further analysis of gene expression in the natural population indicated that *GmRCAβ* accumulated more mRNA in all landraces than *GmRCAα* (Supplementary Fig. S3A), and the levels of *GmRCAα* and *GmRCAβ* expression were significantly different (Supplementary Fig. S3B). Correlation analysis suggested that the expression levels of both genes were positively correlated with Φ_PSII_, qP, and seed yield, but the correlations between *GmRCAβ* expression level and Φ_PSII_, qP, and seed yield were slightly higher than for *GmRCAα*. In addition, *GmRCAβ* expression level displayed a positive correlation with seed weight (Table 2). Overall, these results suggested that soybean landraces exhibited considerable natural variation in RCA gene expression level and that both isoforms could play an important role in modulating soybean photosynthetic capacity and seed yield.

### eQTL analysis of *GmRCAβ* expression

Since RCA genes were positively correlated with chlorophyll fluorescence parameters and seed yield, changes in *RCA* expression at the transcript level may have a potential applicability in breeding for enhanced soybean productivity. The present study analysed the genetic regulation of *GmRCAβ* expression and evaluated the associations with 1142 SNPs by genome-wide association analysis. A total of 13 SNPs were identified as having significant marker-trait associations at *P ≤* 0.01 (–log*P* ≥ 2.00) for the expression of the *GmRCAβ* (Table 3). Here, a *cis*-eQTL was defined as being located within 5Mb of the target gene; otherwise, it was termed as a *trans*-eQTL (Morley *et al.*, 2004; Ghazalpour *et al.*, 2006; Gatti *et al.*, 2009, 2011; Swanson-Wagner *et al.*, 2009; Grundberg *et al.*, 2010). Among the 13 SNPs, BARC-040479-07752 was located in the same chromosome as *GmRCAβ* (chromosome 18) and was 1.62Mb away from *GmRCAβ*, suggesting that this SNP might be a *cis*-eQTL, while BARC-021337-04040 was also located in chromosome 18 and was 7.87Mb away from *GmRCAβ*, suggesting that this SNP might act in *trans*, but *GmRCAβ* was located between the two SNPs. The other 11 SNPs were located in other chromosomes, indicating that they may influence the expression levels of *GmRCAβ* via *trans*-regulation. These results indicated that *GmRCAβ* expression might be controlled by a combination of both *cis*-acting and *trans*-acting eQTL. In addition, the *cis*-acting eQTL that had the stronger association (–log*P* = 5.64) with expression may mainly control the expression of *GmRCAβ*.

**Table 3.** Expression quantitative trait loci mapping of *GmRCAβ* in a natural population

SNP	Chromosome	Position	−log*P*
BARC-032333-08950	14	5 286 848	2.00
BARC-042189-08197	4	43 683 671	2.18
BARC-028177-05786	6	13 550 805	2.64
BARC-028177-05785	6	13 550 805	2.26
BARC-021247-04012	6	47 038 336	2.02
BARC-017541-03068	17	38 843 283	3.06
BARC-025663-04986	15	9 360 685	2.10
BARC-040479-07752	18	1 228 510	5.64
BARC-021337-04040	18	10 714 500	2.32
BARC-019775-04370	12	7 494 368	2.03
BARC-039961-07622	12	15 574 190	2.33
BARC-018911-03272	10	3 519 980	2.74
BARC-031677-07213	10	43 841 452	2.36

Significant association: *P* ≤ 0.01, –log*P* ≥ 2.00. SNP, single-nucleotide polymorphism.

### Allelic variation in the *GmRCAβ* promoter

**Fig. 2.**
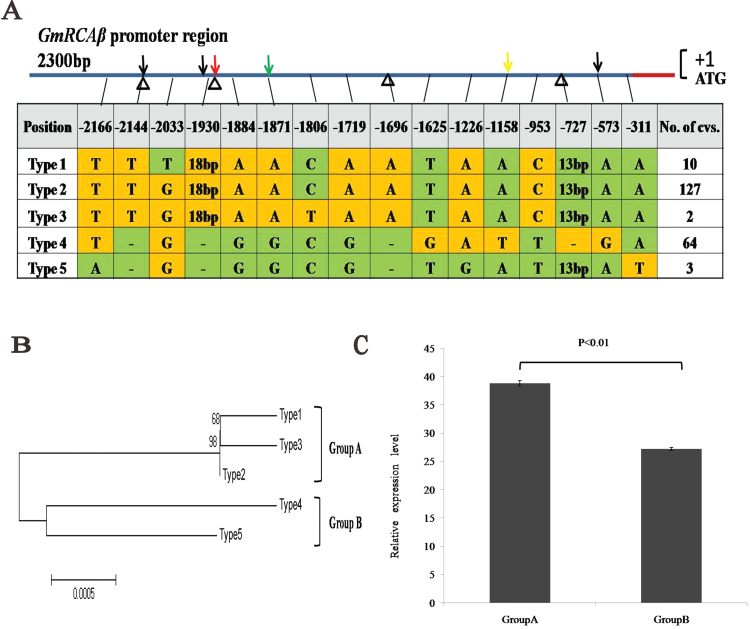
Functional and polymorphic analysis of the *GmRCAβ* promoter region in a natural population of soybean. (A) Nucleotide polymorphisms in the *GmRCAβ* promoter region; polymorphic nucleotides are indicated by different colours, red arrow indicates the AT1 motif, black arrows indicate the TATA boxes, green arrow indicates the ARE, and the yellow arrow indicates the GAG motif; arrowheads indicate deletion and insertion sites. (B) Neighbour-joining phylogenetic analysis of *GmRCAβ* promoter sequences. (C) *GmRCAβ* mRNA levels in landraces with group A and B promoters; leaves were harvested at R6 stage (full seed) and analysed by quantitative real-time PCR; error bars represent standard errors of three independent repetitions (this figure is available in colour at *JXB* online).

As the expression level of *GmRCAβ* is the result of interactions of multiple *cis*-and *trans*-acting genetic factors, and because *cis*-acting eQTLs may mainly control *GmRCAβ* expression, the *GmRCAβ* promoter sequence, which may affect both the *cis*- and *trans*-acting eQTLs (Vuylsteke and van Eeuwijk, 2008), might be a potential factor in expression level diversity. To identify whether sequence polymorphisms in the promoter region of *GmRCAβ* were related to the observed expression level, a 2300-bp region of the promoter, which was shown to be sufficient for the proper regulation of expression in plant (Orozco and Ogren, 1993; Liu *et al.*, 1996; Qu *et al.*, 2011; Yang *et al.*, 2012), was cloned and sequenced, including the promoter and the 5′-UTR region, in a natural population including 219 landraces of different geographic origins (Supplementary Figs S4 and S5). Multiple sequence alignment revealed 16 polymorphic sites and five types of sequences (Fig. 2A). Phylogenetic trees were constructed using neighbour-joining and Bayesian approaches (Fig. 2B and Supplementary Fig. S6), which indicated that these sequence types were classified into two groups: types 1, 2, and 3 in group A and types 4 and 5 in group B. Genes with group A promoters were expressed at significantly higher levels than those with group B promoters (Fig. 2C), suggesting an association between promoter sequence type and expression level. Of these polymorphisms, five polymorphic sites, including SNPs and indels, were located in core regulatory elements such as the GAG motif, the AT-1 motif, and the TATA box (Table 4).

**Table 4.** Polymorphic sites in the promoter region of *GmRCAβ*

Position	Polymorphic type	Regulatory element change	Function of *cis*-elements	Frequency (%)
–2166	T/A	–	–	1.46
–2144	1-base indel	TATA box	Core promoter element around –30 of transcription start	67.48
–2033	T/G	–	–	4.85
–1930	18-base indel	AT1 motif	Part of a light-responsive module	67.48
		TATA box	Core promoter element around –30 of transcription start	
–1884	A/G	–	–	67.48
–1871	A/G	ARE	*cis*-Acting regulatory element essential for anaerobic induction	67.48
–1806	C/T	–	–	0.97
–1719	A/G	–	–	67.48
–1696	1-base indel	–	–	67.48
–1625	T/G	–	–	68.93
–1226	A/G	–	–	1.46
–1158	A/T	GAG motif	Part of a light responsive element	68.93
–953	C/T	–	–	67.48
–727	13-base indel	–	–	68.93
–573	A/G	TATA box	Core promoter element around –30 of transcription start	68.93
–311	A/T	–	–	1.46

–, not located in regulatory elements.

### Candidate-gene association analysis of the SNPs in the promoter region of *GmRCAβ*

To validate whether the sequence polymorphisms in the promoter region were the causal sites for expression level variation of *GmRCAβ*, candidate-gene association analysis was performed. All SNPs with a minor allele frequency above 10% were considered. Significantly associated SNPs (including indels) were identified by EM analysis (expectation-maximization, an algorithm for solving mixed models in association analysis; Yu *et al.*, 2005) using TASSEL software. For *GmRCAβ*, there were seven SNPs (including an 18-bp indel) significantly associated with the expression of *GmRCAβ* (Table 5). Interestingly, three SNPs were located in the regulatory elements respectively, such as the AT-1 motif, the TATA box, and the anaerobic induction-responsive element. In addition, all associated SNPs existed in a linked manner (in the same linkage disequilibrium block) with the same *P*-value.

**Table 5.** Single-nucleotide polymorphisms in the *GmRCAβ* promotor region significantly associated with gene expression

Position	Polymorphic type	Regulatory element change
–2144	1-base indel	TATA box
–1930	18-base indel	AT1 motif, TATA box
–1884	A/G	–
–1871	A/G	ARE
–1719	A/G	–
–1696	1-base indel	–
–953	C/T	–

All significant associations: *P* = 1.38. –, not located in regulatory elements.

### *GUS* expression driven by different promoter sequence types

**Fig. 3.**
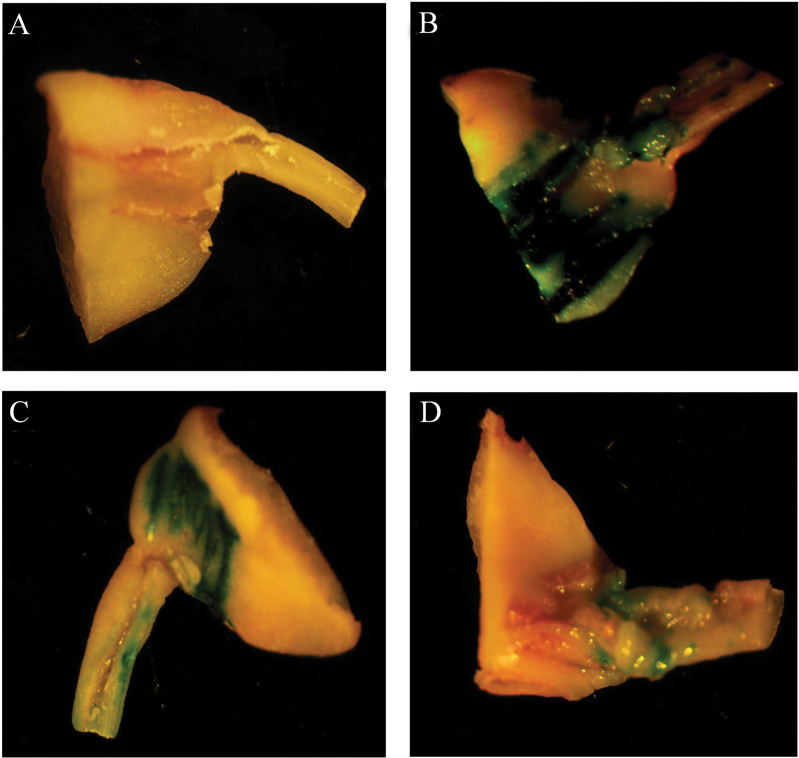
Histochemical staining of soybean cotyledonary nodes driven by the different promoter sequence types of *GmRCAβ*: (A) promoterless control; (B) CaMV 35S promoter; (C) group A type promoter; (D) group B type promoter (this figure is available in colour at *JXB* online).

Although several SNPs in the promoter region of *GmRCAβ* were identified and associated with the expression of *GmRCAβ*, these SNPs were mainly classified into two groups. Therefore, GUS expression behind group A and group B promoters was compared using a transient assay to determine whether these different promoter types could result in differences in expression level. The type 2 promoter sequence of group A and type 4 promoter sequence of group B were cloned (Supplementary Fig. S7A) and fused with the GUS gene as a reporter (Supplementary Fig. S7B). Histochemical staining indicated that both group A and group B promoters were able to drive the GUS expression, but GUS expression driven by the group A promoter was stronger than that by the group B promoter (Fig. 3). These results indicated that the promoter sequence type of *GmRCAβ* could influence gene expression level.

## Discussion

### Different *RCA* isoform expression patterns in soybean

In most plants, the expression of the RCA gene is tissue specific and light inducible and exhibits diurnal and circadian oscillations in addition to being affected by development and phytohormones (Zielinski *et al.*, 1989; Pilgrim and McClung, 1993; Zhang and Komatsu, 2000; Sharma and Komatsu, 2002). In this study, expression pattern analysis of *RCA* indicated that *GmRCAα* and *GmRCAβ* in soybean were organ specific and followed a diurnal pattern (Fig. 1A, B) but that their expression levels were significantly different (Fig. 1A and Supplementary Fig. S3A). Consistent with these results, soybean leaves contained unequal amounts of the two isoforms (Fig. 1C), with higher levels of the small isoform (Salvucci *et al.*, 1987). It is not clear why the different patterns of RCA isoform expression in soybean and other species such as rice and wheat result in the accumulation of a greater amount of β-RCA than α-RCA, whereas certain plant species such as *Arabidopsis*, camelinam and spinach express equal amounts of α- and β-RCA (Salvucci *et al.*, 1987; Wang *et al.*, 2010; Fukayama *et al.*, 2012). Recent studies on species differences in the regulatory properties of RCA have indicated that the redox regulation of α-RCA, which provides fine control over the sensitivity of the holoenzyme to inhibition by ADP, does not seem to be essential in all plant species. Similarly, ADP inhibition of β-RCA varies among plant species and even occurs in species that also express a α-RCA (Carmo-Silva and Salvucci, 2013). Hence, the different patterns of RCA isoform expression observed in the current study may be necessary for control over enzyme activity and increasing overall soybean photosynthesis and productivity.

What causes the significantly different expression levels of the two RCA genes in soybean? This study compared the sequences of the two promoters, which play a key role in gene expression and regulation. Some *cis*-elements were specifically present in their promoter region, and the copy numbers of each *cis*-element were different between two RCA genes (Table 1). It has been reported that promoter inducibility and strength varies depending on *cis*-element copy numbers and, more specifically, spacing of motifs relative to the TATA box (Rushton *et al.*, 2002; Gurr and Rushton, 2005). The differences in copy numbers of some important *cis*-elements, such as G-box and pyrimidine-rich 5′-UTR, might cause different expression of the two RCA genes. In addition, I-box elements, which have been shown to be involved in light-regulated and/or circadian clock-regulated expression of photosynthetic genes (Borello *et al.*, 1993; Terzaghi and Cashmore, 1995; Agius *et al.*, 2005), were only present in *GmRCAα*. However, it has been demonstrated that I-box elements downregulate gene expression in strawberry and melon fruit, suggesting a new function of the I-box elements as a negative regulator (Yamagata *et al.*, 2002; Agius *et al.*, 2005).

### Expression levels of *GmRCAα* and *GmRCAβ* may modulate seed yield

Although different patterns of RCA gene expression were observed in soybean, the effect of this on yield components remains unknown. A direct link between growth and gene expression has been confirmed in rice, in which different levels of expression that are positively correlated with these traits of this agronomically important grain are dependent on sequence variation in the promoter of the GS5 gene (Li *et al.*, 2011). Studies have shown that endogenous levels of *RCA* expression are of importance to plant photosynthesis under both optimal (Martínez-Barajas *et al.*, 1997) and supraoptimal temperatures (Ristic *et al.*, 2009). Thus, the present study investigated the effect of expression on soybean yield by evaluating the expression levels of *RCA* in a natural population. The expression levels of both genes were positively correlated with chlorophyll fluorescence parameters and seed yield, suggesting that changes in *RCA* expression has a potential applicability in breeding for enhanced soybean productivity. Moreover, the expression levels displayed high diversity in the germplasm, and the phenotypic values in natural population ranged from 0.47 to 66.41 for *GmRCAα* expression and from 2.38 to 196.49 for *GmRCAβ* expression. However, the regression analysis indicated that a given change in *GmRCAα* was associated with a greater change in photosynthesis or yield (Supplementary Table S2). Therefore, further studies need to compare *GmRCAα* and *GmRCAβ* in transgenic soybean.

### New insights into the genetic regulation of *GmRCAβ* expression

Gene expression is believed to be a composite reflection of multiple genetic *cis*- or *trans*-acting factors, according to eQTL analysis (Delker and Quint, 2011). Moreover, target gene expression can be controlled by a combination of both *cis*- and *trans*-acting elements (Druka *et al.*, 2010). A previous study using a recombinant inbred line of soybean detected only two *trans*-acting eQTLs for *GmRCAβ* (Yin *et al.*, 2010b), suggesting that expression of this gene might be a consequence of *trans*-regulation. However, it must be emphasized that all QTL studies rely on natural genetic variation in the population under study (Druka *et al.*, 2010). Only those eQTLs detected in different materials or under multiple environments are valuable for breeding (Yin *et al.*, 2010b). In the present study, further eQTL mapping was conducted in a collection of 219 soybean landraces that represented the genetic diversity of landraces from different geographic origins in China (Supplementary Fig. S1). The eQTL mapping indicated that the expression of *GmRCAβ* could be controlled by a combination of both *cis*-acting and *trans*-acting eQTLs. In agreement with this finding, the complex expression pattern of *RCA* reflects the interactions of multiple *trans*-acting protein factors with multiple cognate *cis*-acting DNA elements (Liu *et al.*, 1996). However, no coincident eQTLs were observed between the current and the previous eQTL study (Yin *et al.*, 2010b). A possible reason for this might be the differences in the mapping populations, since the natural population has higher diversity and has undergone natural selection in the process of evolution, which may be more favourable for QTL mapping of the minor or larger effects. Other possible reasons might be the low marker density, phenotyping error, or statistical defects associated with genome-wide association analysis (Zhao *et al.*, 2011). In future studies for further understanding the genetic mechanisms of the expression levels of *GmRCAβ* in soybean, more diverse germplasms with lower linkage disequilibrium and more candidate gene-based markers will be chosen to detect the possible causal genes.

Although this study identified multiple eQTLs that were responsible for expression variation, the mechanism of how gene expression is regulated by eQTLs remains unknown. The promoter of *GmRCAβ* that affects both *cis*-acting and *trans*-acting eQTLs (Vuylsteke and van Eeuwijk, 2008) might be a potential factor generating diversity at the expression level. Moreover, it is well known that promoter elements play a key role in gene regulation and any changes in their sequences will dramatically influence gene expression resulting in variable trait expression. In the current study, the SNPs in the *GmRCAβ* promoter were identified and shown to correlate with expression level. Further transient expression showed that GUS expression driven by the group A promoter was stronger than that by the group B promoter, suggesting that promoter sequence types could influence the expression level of *GmRCAβ*. A similar result has been observed in rice, in which the type of *Hd3a* promoter contributed to diversity in flowering time and *Hd3a* expression level (Takahashi *et al.*, 2009).

However, such diversity of expression level may not be solely determined by promoter sequence, because introns and 3′-UTRs also have significant effect on transcript synthesis and accumulation, which in turn alter the trait expression (Kumar *et al.*, 2010). DeRidder *et al.* (2012) recently showed that *AtRCAβ* transcripts lacking their native 3′-UTR were significantly more stable than their full-length counterparts, indicating post-transcriptional regulation might participate in controlling of *RCA* expression in *Arabidopsis*. In addition, acclimation of *RCA* expression in *Arabidopsis* appeared to be due to changes in mRNA stability, not to increased rates of transcription. Hence, changes in mRNA levels may be the result either from transcriptional or post-transcriptional regulation, or both.

### SNPs associated with *GmRCAβ* expression and their applications for crop improvement

The identification of alleles that affect key phenotypes is of utmost importance for the utilization of genetic resources in crop improvement (Holloway and Li, 2010). In human, more than 100 *cis*-regulatory polymorphisms were identified and shown to associate with phenotypic variation (Wray, 2007). In the present study, seven SNPs were identified as significantly associated with the expression of *GmRCAβ* (Table 5). Interestingly, three SNPs were located in regulatory elements such as the AT1 motif and the TATA box, which are important for the light regulation of gene expression, and data gathered in several species indicate that the expression of *RCA* is highly regulated by light (Qu *et al.*, 2011). These SNPs in regulatory regions may impact the phenotype by altering the activity of the promoter and changing the expression levels of the RCA genes (Kumar *et al.*, 2010). In addition, all associated SNPs existed in a linked manner (in the same linkage disequilibrium block) with the same *P*-value, indicating that any of the SNPs can be used as an allele-specific molecular marker (Kumar *et al.*, 2010) to optimize gene expression. The identification of these allele-specific molecular markers would improve the understanding of how variation within regulatory factors affects transcript accumulation, enabling the prediction of the mechanisms by which genetic variation within these genetic elements shapes phenotypic diversity in natural populations, which would, in turn, facilitate the introgression of novel alleles through marker-assisted breeding.

## Supplementary material

Supplementary data are available at *JXB* online.

Supplementary Fig. S1. The geographic distributions of landraces used in this study

Supplementary Fig. S2. Multiple sequence alignment of promoter sequences of *GmRCAα* and *GmRCAβ*.

Supplementary Fig. S3. Expression levels of *GmRCAα* and *GmRCAβ* in the natural population.

Supplementary Fig. S4. Sequence analysis of the *GmRCAβ* promoter in soybean.

Supplementary Fig. S5. Sequence analysis of the *GmRCAα* promoter in soybean.

Supplementary Fig. S6. Phylogenetic analysis of *GmRCAβ* promoter sequence.

Supplementary Fig. S7. PCR products and restriction analysis of 1381Z group A and group B promoters.

Supplementary Table S1. Descriptive statistics and variance analysis of *GmRCAα* and *GmRCAβ* expression in natural population.

Supplementary Table S2. Regression coefficients among *GmRCAα* and *GmRCAβ* expression and yield components in a natural soybean population.

Supplementary Table S3. Putative *cis*-acting elements and functions for *GmRCAβ*.

Supplementary Table S4. Putative *cis*-acting elements and functions for *GmRCAα*.

Supplementary Data
