# Supplementary material for: Variation in Rubisco activase (RCAβ) gene promoters and expression in soybean [Glycine max (L.) Merr.]
Source: J Exp Bot. 2013 Oct 29;65(1):47–59. doi: 10.1093/jxb/ert346 (PMC3883283; doi:10.1093/jxb/ert346)
Supplement: Supplementary Data [file supp_65_1_47__index.html]

Variation in Rubisco activase (RCAβ) gene promoters and expression in soybean [Glycine max (L.) Merr.] — Supplementary Data 

# Variation in Rubisco activase (*RCAβ*) gene promoters and expression in soybean [*Glycine max* (L.) Merr.]

## Supplementary Data

Data files

**Files in this Data Supplement:**

- Supplementary Data - Supplementary Data
