# Supplementary material for: Variation in Rubisco activase (RCAβ) gene promoters and expression in soybean [Glycine max (L.) Merr.]
Source: J Exp Bot. 2013 Oct 29;65(1):47–59. doi: 10.1093/jxb/ert346 (PMC3883283; doi:10.1093/jxb/ert346)
Supplement: Supplementary Data [file supp_ert346_ert346_supplementary.pdf]

Supplementary Data

Figure Legends

Figure S1. The geographic distributions of landraces used in this study. Each circle represents a different province. The number of landraces per province is indicated by closed portion of the circle. The four colored portions divide China into four regions: NER (northeast region), NR (north region), HR (Huanghuai region) and SR (south region).

Figure S2. Multiple sequence alignment result of promoter sequences of *GmRCAα* and *GmRCAβ* by ClustalX v.1.83 program. A 2300-bp fragment upstream of the ATG start codon was designated as the promoter region.

Figure S3. The expression levels of two RCA genes in the natural population. A. The distribution of the expression levels of two RCA genes in each landrace. B. A comparison of the average value of two RCA genes in 219 soybean landraces.

Figure S4. Sequence analysis of the *GmRCAβ* promoter in soybean. A 2300-bp fragment upstream of the ATG start codon of *GmRCAβ* was designated as the promoter region. Parts of the putative cis-regulatory elements are noted under the sequences in shadow letters. The ATG is indicated in red. The nucleotide at position +1 (bold A in the gray box) is the ATG start codon. The polymorphism sites are underlined, and the cis-acting elements located in polymorphism sites are indicated in yellow.

Figure S5. Sequence analysis of the *GmRCAα* promoter in soybean. A 2300-bp fragment upstream of the ATG start codon of *GmRCAα* was designated as the promoter region. Parts of the putative cis-regulatory elements are noted under the sequences in shadow letters. The ATG is indicated in red. The nucleotide at position +1 (bold A in the gray box) is the ATG start codon.

Figure S6. Phylogenetic analysis of *GmRCAβ* promoter sequence. The tree was constructed with the Bayesian approaches using MrBayes v3.1.

Figure S7. PCR product and restriction analysis of 1381Z-GroupA promoter,1381Z-GroupB promoter. A. Amplified products of genomic DNA of different *GmRCAβ* promoter sequence in soybean. B. Vector pCAMBIA1381Z restriction results.

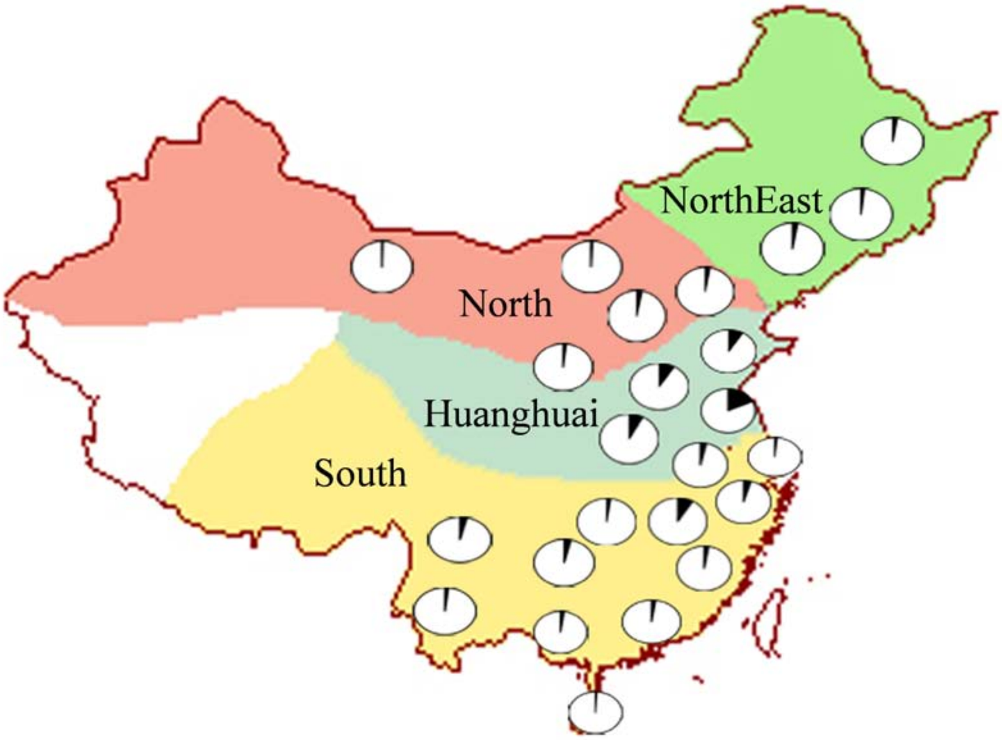

Figure S1. The geographic distributions of landraces used in this study. Each circle represents a different province. The number of landraces per province is indicated by closed portion of the circle. The four colored portions divide China into four regions: NER (northeast region), NR (north region), HR (Huanghuai region) and SR (south region).

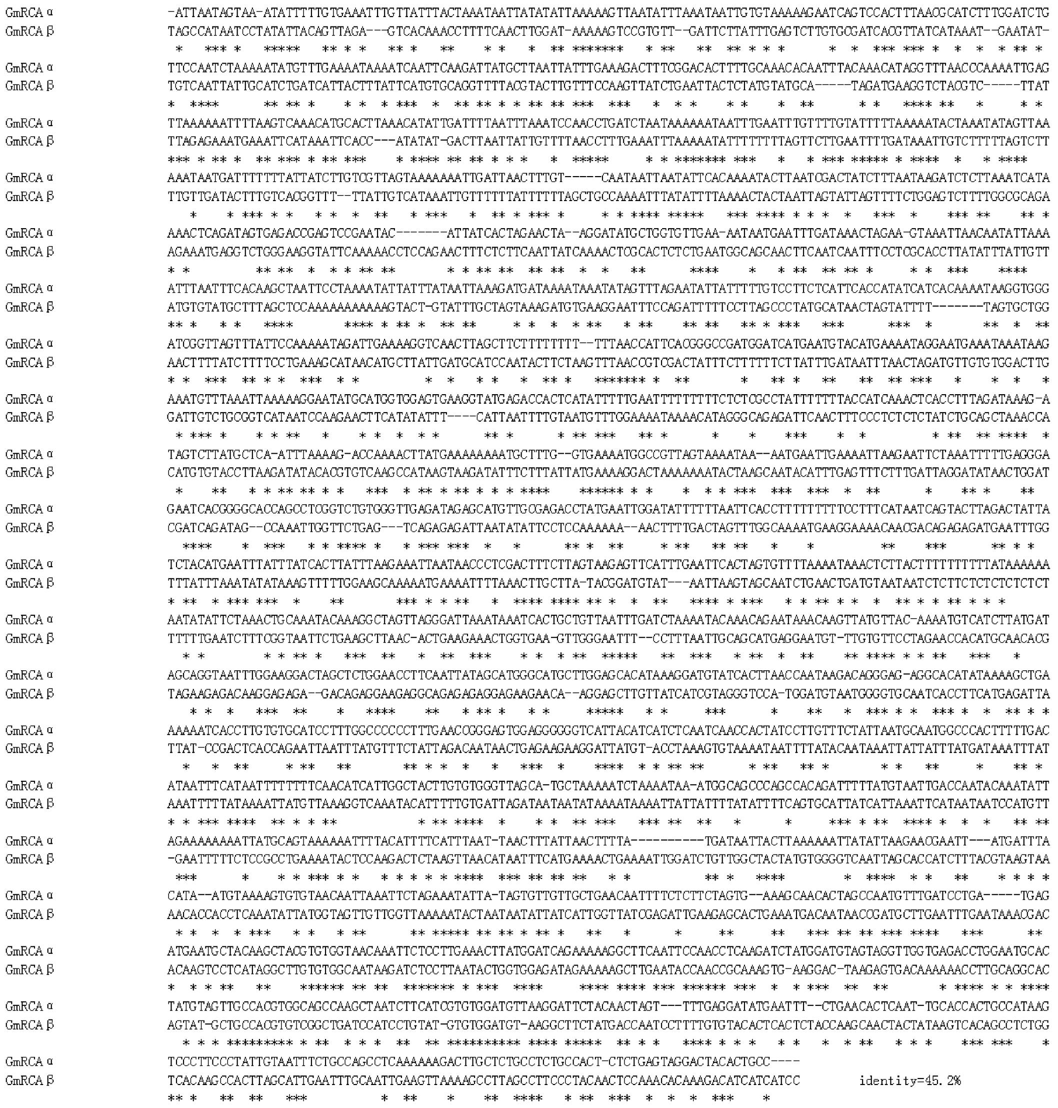

Figure S2. Multiple sequence alignment result of promoter sequences of *GmRCAα* and *GmRCAβ* by ClustalX v.1.83 program. A 2300-bp fragment upstream of the ATG start codon was designated as the promoter region.

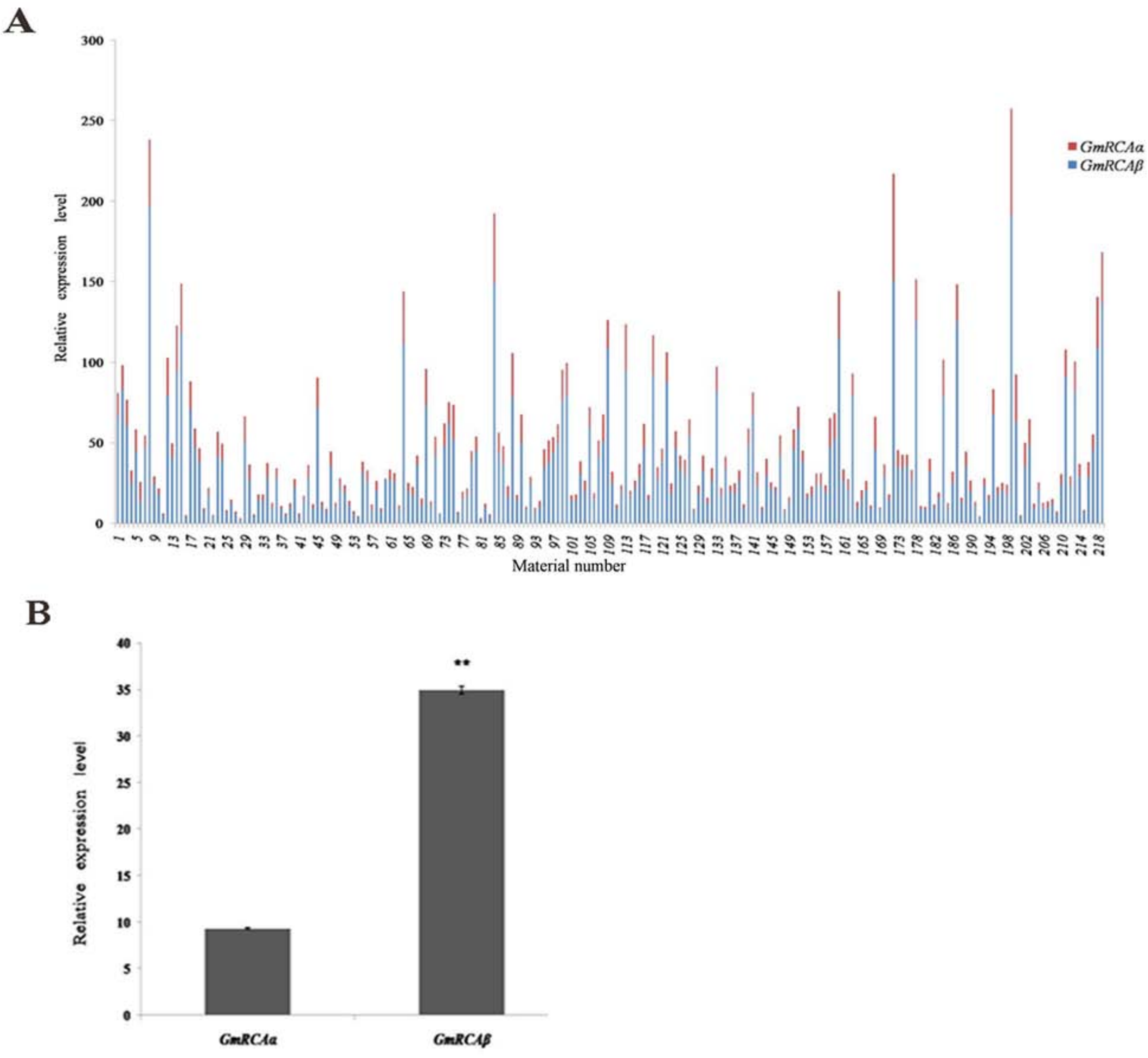

Figure S3. The expression levels of two RCA genes in the natural population. A. The distribution of the expression levels of two RCA genes in each landraces. B. A comparison of the average value of two RCA genes in 219 soybean landraces.

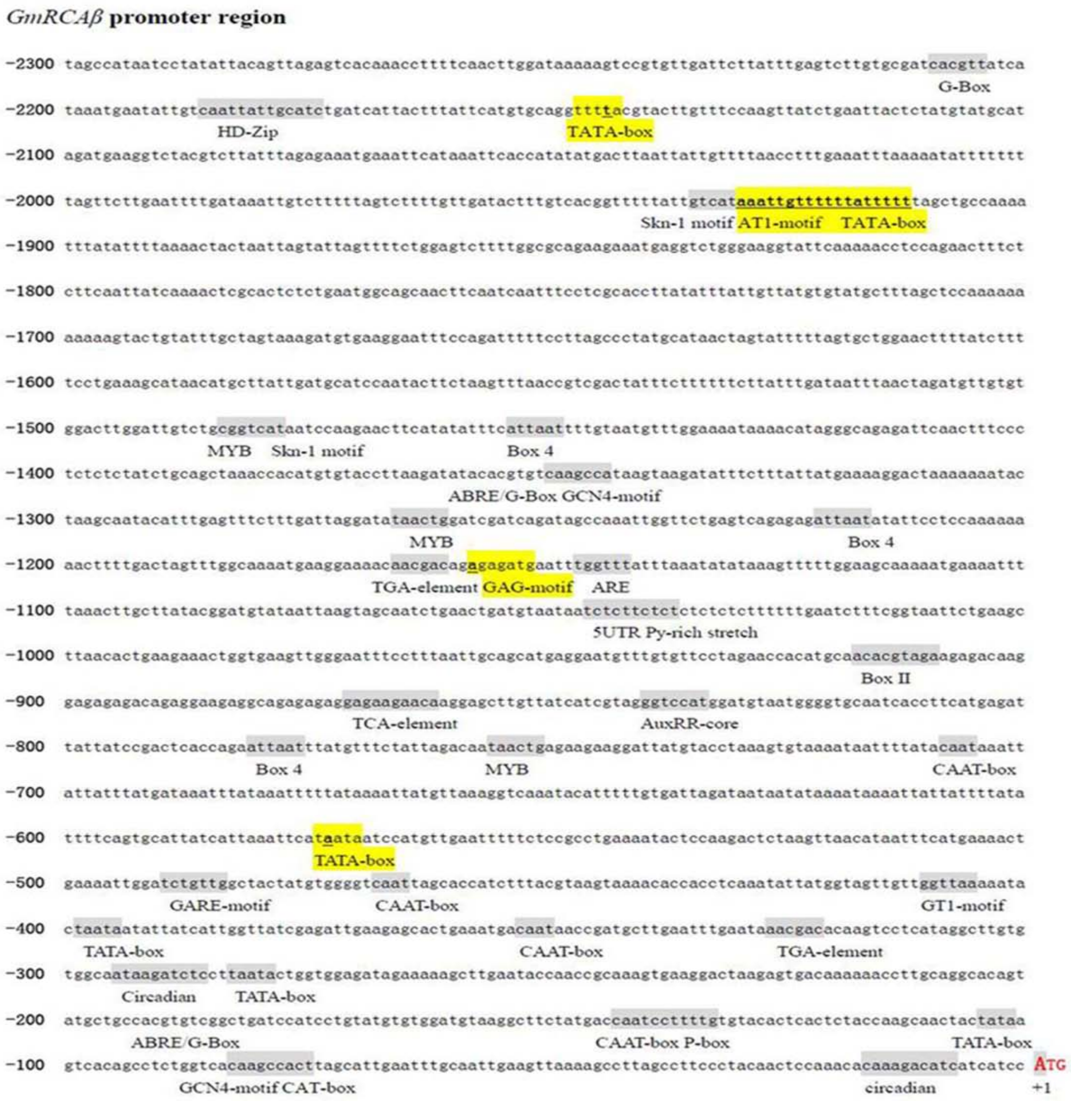

Figure S4. Sequence analysis of the *GmRCAβ* promoter in soybean. A 2300-bp fragment upstream of the ATG start codon of *GmRCAβ* was designated as the promoter region. Parts of the putative cis-regulatory elements are noted under the sequences in shadow letters. The ATG is indicated in red. The nucleotide at position +1 (bold A in the gray box) is the ATG start codon. The polymorphism sites are underlined, and the cis-acting elements located in polymorphism sites are indicated in yellow.

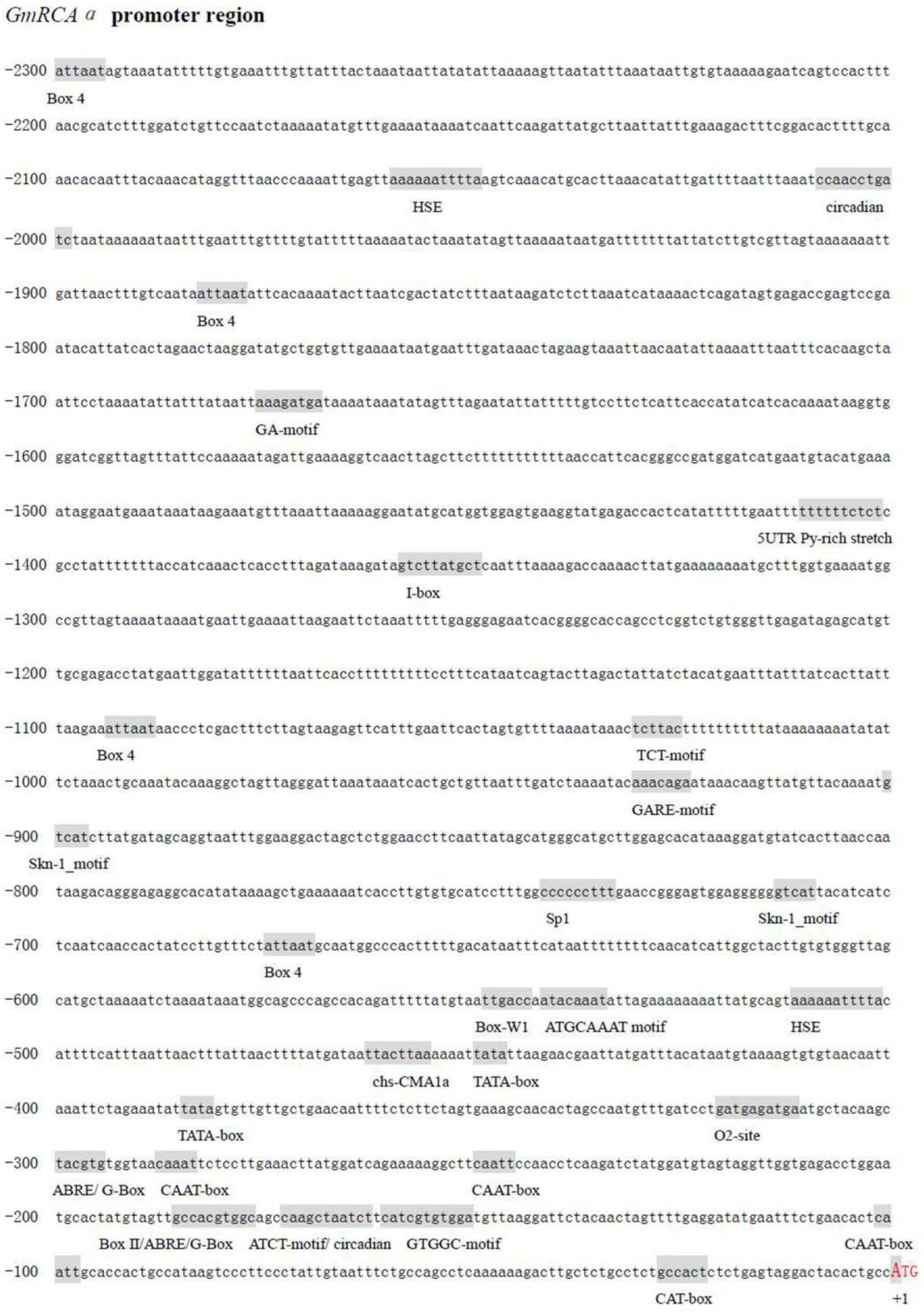

Figure S5. Sequence analysis of the *GmRCAα* promoter in soybean. A 2300-bp fragment upstream of the ATG start codon of *GmRCAα* was designated as the promoter region. Parts of the putative cis-regulatory elements are noted under the sequences in shadow letters. The ATG is indicated in red. The nucleotide at position +1 (bold A in the gray box) is the ATG start codon.

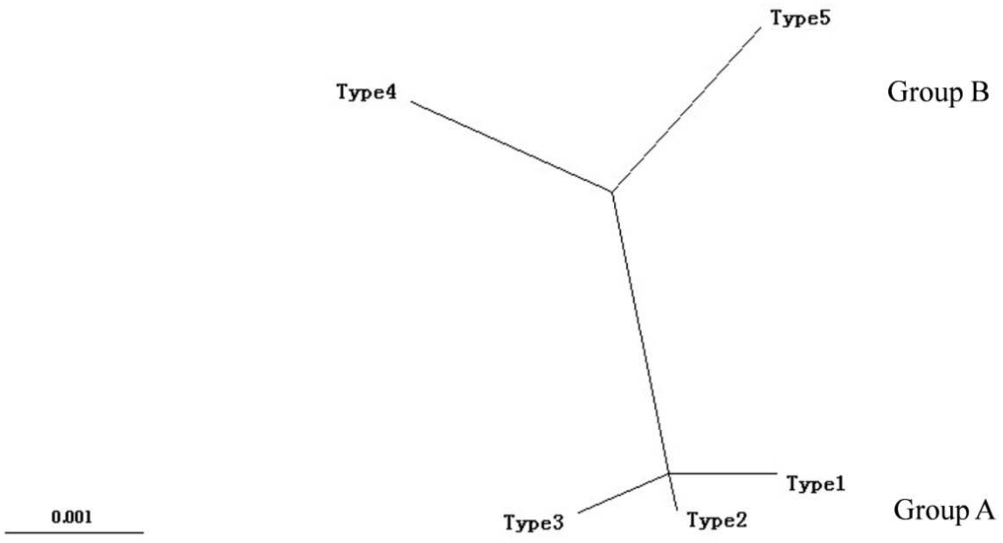

Figure S6. Phylogenetic analysis of *GmRCAβ* promoter sequence. The tree was constructed with the Bayesian approaches using MrBayes v3.1.

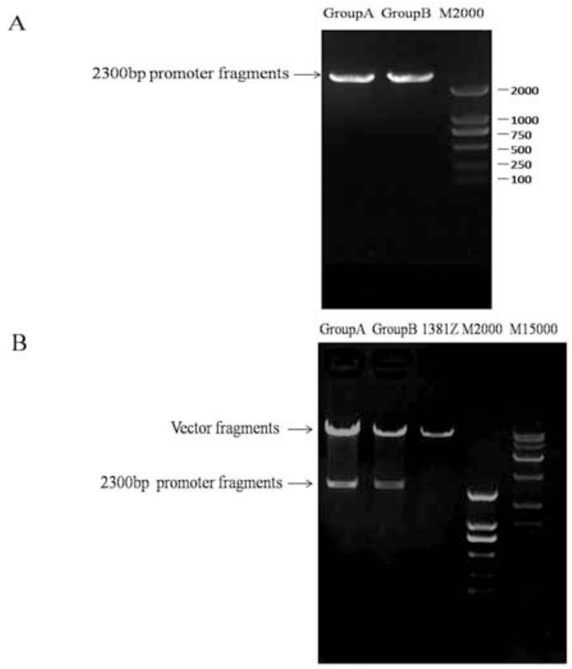

Figure S7. PCR product and restriction analysis of 1381Z-GroupA promoter,1381Z-GroupB promoter. A. Amplified products of genomic DNA of soybean different *GmRCAβ* promoter sequence. B. Vector pCAMBIA1381Z restriction results.

Table S1. The descriptive statistics and variance analysis of *GmRCAa* expression and *GmRCAβ* expression in natural population

Traits	Descriptive statistics			Variance analysis				
	Mean	Range	SD	Source of variation	df	Sum of square	Mean of square	P value
GmRCAα expression	9.26	0.47-66.41	9.6	Among landraces	217	60336.78	278.05	<0.001
				Block(time segment)	2	3.32	1.66	<0.05
				Error	434	171.77	0.4	
				Total	653	60511.87	280.11	
GmRCAβ expression	34.65	2.38-196.49	33.36	Among landraces	217	742218.73	3420.36	<0.001
				Block(time segment)	2	73.8	36.9	<0.001
				Error	434	2086.55	4.81	
				Total	653	744379.08	3462.07	

Table S2. Regression coefficients among *GmRCAα* expression, *GmRCAβ* expression and yield components in a natural soybean population

Traits	Fv/Fm [a]	Φ_{PSII} [b]	qP [c]	NPQ [d]	Seed weight	Seed yield
GmRCAβ expression	ns	0.0003*	0.0004**	ns	0.027*	0.025**
GmRCAα expression	ns	0.001*	0.001**	ns	ns	0.073**

[a] Fv/Fm: maximum quantum yield of PSII primary photochemistry in the dark-adapted state; [b] ΦPSII: actual quantum yield in the light-adapted state; [c] qP: photochemical quenching coefficient; [d] NPQ: non-photochemical quenching parameter describing the regulated dissipation of excess energy; ns: not significant; *, $P<0.05$; **, $P<0.01$.

Table S3 The putative cis-acting elements and functions predicted by PlantCARE in the *GmRCAβ* promoter

Elements type	Names of the elements	Organism	Motif sequence	Function
Light	AT1-motif	*Solanum tuberosum*	AATTATTTTTTATT	part of a light-responsive module
	Box 4	*Petroselinum crispum*	ATTAAT	part of a conserved DNA module involved in light responsiveness
	Box II	*Arabidopsis thaliana*	ACACGTAGA	part of a light-responsive element
	GAG-motif	*Spinacia oleracea*	AGAGATG	part of a light-responsive element
	GT1-motif	*Arabidopsis thaliana*	GGTTAA	light-responsive element
	G-Box	*Pisum sativum*	CACGTT	cis-acting regulatory element involved in light responsiveness
		Brassica napus	ACACGTGT	
		Antirrhinum majus	CACGTA	
		Arabidopsis thaliana	CACGTG	
Circadian	circadian	*Lycopersicon esculentum*	CAAAGATATC	cis-acting regulatory element involved in circadian control
Phytohormone	ABRE	*Arabidopsis thaliana*	CACGTG	cis-acting element involved in the abscisic acid responsiveness
	AuxRR-core	*Nicotiana tabacum*	GGTCCAT	cis-acting regulatory element involved in auxin responsiveness
	TGA-element	*Brassica oleracea*	AACGAC	auxin-responsive element
	TCA-element	*Brassica oleracea*	GAGAAGAATA	cis-acting element involved in salicylic acid responsiveness
	P-box	*Oryza sativa*	CCTTTTG	gibberellin-responsive element
	GARE-motif	*Brassica oleracea*	TCTGTTG	gibberellin-responsive element
Basal element	CAAT-box	*Glycine max*	CAATT	common cis-acting element in promoter and enhancer regions
		Hordeum vulgare	CAAT	
		Arabidopsis thaliana	CCAAT	
	TATA-box	*Arabidopsis thaliana*	TATA	core promoter element around -30 of transcription start
		Brassica napus	ATATAT	
		Lycopersicon esculentum	TTTTA	
	5UTR Py-rich stretch	*Lycopersicon esculentum*	TTTCTTCTCT	cis-acting element conferring high transcription levels
Biotic stress	MBS	*Zea mays*	CGGTCA	MYB binding site
		Arabidopsis thaliana	TAACTG	MYB binding site involved in drought-inducibility
	ARE	*Zea mays*	TGGTTT	cis-acting regulatory element essential for the anaerobic induction
Other	Skn-1_motif	*Oryza sativa*	GTCAT	cis-acting regulatory element required for endosperm expression
	CAT-box	*Arabidopsis thaliana*	GCCACT	cis-acting regulatory element related to meristem expression
	GCN4_motif	*Oryza sativa*	CAAGCCA	cis-regulatory element involved in endosperm expression
	HD-Zip	*Arabidopsis thaliana*	CAAT(A/T)ATTG	element involved in the differentiation of the palisade mesophyll cells
			CAAT(G/C)ATTG	element involved in the control of leaf morphology development

Table S4. The putative cis-acting elements and functions predicted by PlantCARE in the *GmRCAa* promoter

Elements type	Names of the elements	Organism	Motif sequence	Function
Light	GA-motif:	*Helianthus annuus*	AAAGATGA	part of a light responsive element
	I-box	*Nicotiana plumbaginifolia*	CTCTTATGCT	part of a light responsive element
		Solanum tuberosum	TATTATCTAGA	
	ATCT-motif	*Arabidopsis thaliana*	AATCTAATCT	part of a conserved DNA module involved in light responsiveness
	Box II	*Petroselinum crispum*	CCACGTGGC	part of a light responsive element
	Sp1	*Zea mays*	CC(G/A)CCC	light responsive element
	TCT-motif	*Arabidopsis thaliana*	TCTTAC	part of a light responsive element
	chs-CMA1a	*Daucus carota*	TTACTTAA	part of a light responsive element
	GTGGC-motif	*Arabidopsis thaliana*	CATCGTGTGGC	part of a light responsive element
	Box 4	*Petroselinum crispum*	ATTAAT	part of a conserved DNA module involved in light responsiveness
	G-Box	*Pisum sativum*	CACGTG	cis-acting regulatory element involved in light responsiveness
		Daucus carota	TACGTG	
		Arabidopsis thaliana	CACGTG	
Circadian	circadian	*Lycopersicon esculentum*	CAAAGATATC	cis-acting regulatory element involved in circadian control
Phytohormone	ABRE	*Arabidopsis thaliana*	TACGTG	cis-acting element involved in the abscisic acid responsiveness
			CACGTG	
			ACGTGGC	
	GARE-motif	*Brassica oleracea*	AAACAGA	gibberellin-responsive element
Biotic stress	HSE	*Brassica oleracea*	AAAAAATTTC	cis-acting element involved in heat stress responsiveness
	Box-W1	*Petroselinum crispum*	TTGACC	fungal elicitor responsive element
Basal element	CAAT-box	*Glycine max*	CAATT	common cis-acting element in promoter and enhancer regions
		Hordeum vulgare	CAAT	
		Arabidopsis thaliana	CCAAT	
	TATA-box	*Arabidopsis thaliana*	TATA	core promoter element around -30 of transcription start
		Brassica napus	ATATAT	
		Lycopersicon esculentum	TTTTA	
	5UTR Py-rich stretch	*Lycopersicon esculentum*	TTTCTTCTCT	cis-acting element conferring high transcription levels
Other	ATGCAAAT motif	*Oryza sativa*	ATACAAAT	cis-acting regulatory element associated to the TGAGTCA motif
	CAT-box	*Arabidopsis thaliana*	GCCACT	cis-acting regulatory element related to meristem expression
	O2-site	*Zea mays*	GATGACATGA	cis-acting regulatory element involved in zein metabolism regulation
	Skn-1_motif	*Oryza sativa*	GTCAT	cis-acting regulatory element required for endosperm expression
